# Mangana Water

**Published:** 1885-03

**Authors:** E. Long Fox


### MANGANA WATER. By E. LONG FOX, M.D.

The manganesian waters of Cransac have been famed as a curative agent since A.D. 900. The village of Cransac is situated in a valley in a mountainous region of the department of Aveyron, in the South of France. The waters were chiefly noted for the large amount of sulphate of manganese contained in them. They were mainly useful in chronic non-inflammatory disorders of the alimentary canal, especially those of an hepatic origin, and in various forms of amenorrhœa. Dr. Bras, physician to the chief hospital of Villefranche, reports the cure by these waters of enormous chronic enlargements of the liver and spleen in soldiers returned from Africa. Within a recent period, however, the nature of the constituents of these waters seems to have changed, and they now contain merely traces of manganese.

In the course of last year it suggested itself to Dr. Griffin, analytical chemist of this city, to form an imitation of these waters as they existed in former times, and to combine the important ingredients with aërated water, at once to make the combination more wholesome and more pleasant. Mr. Keevill, of York Buildings, Clifton, has followed out his suggestions, and now supplies under the name of "Mangana Water" an effervescing mixture of this composition :—

Manganesii Sulph., gr. vii. ss.
Ammonii Chloridi, gr. xv.
Tinct. *Zingiberis*, ♏ xv.
Aquæ Flor. Aurantii, ♏ x.
Aquæ c. $CO^2$ ad ℥ x.

It would not be becoming in me to say anything that might seem to be a testimonial of an article of commerce; but I venture to ask my professional brethren to give some trial to this convenient therapeutical agent, especially in chronic congestion of the liver, and I think they will agree with me in finding it useful. The Mangana Water, like the Cransac springs, would be contra-indicated in acute phthisis, acute internal inflammatory disorder, and in febrile conditions generally.

